# How Taiwan, a non-WHO member, takes actions in response to COVID-19

**DOI:** 10.7189/jogh.10.010380

**Published:** 2020-06

**Authors:** Sheng-Fang Su, Yueh-Ying Han

**Affiliations:** 1 Graduate Institute of Oncology, National Taiwan University College of Medicine, Taipei, Taiwan; 2 YongLin Institute of Health, National Taiwan University, Taipei, Taiwan; 3 Division of Pediatric Pulmonary Medicine, UPMC Children’s Hospital of Pittsburgh, University of Pittsburgh, Pittsburgh, Pennsylvania, USA

On 11 March 2020, the World Health Organization (WHO) declared that the coronavirus disease 2019 (COVID-19) outbreak could be characterized as a pandemic [1]. Meanwhile, COVID-19 has spread rapidly from Wuhan, China to numbers of countries in Asia, the Middle East, and Europe. As of 12 March 2020, more than 100 counties, territories or areas had reported COVID-19 cases with a total of 125 260 confirmed cases and 4613 deaths globally [1,2].

## WORLDWIDE PANDEMIC TOP RECORD

**Figure 1:**
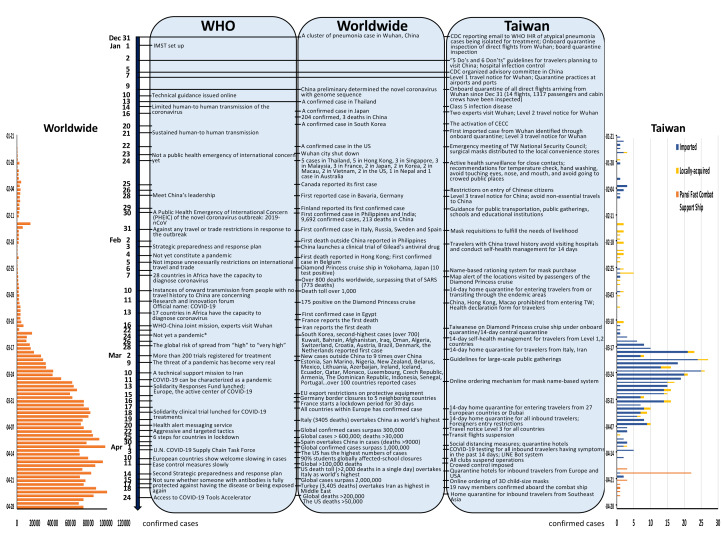
Summary of COVID-19 outbreak- WHO, worldwide, and Taiwan. IMST - incident Management Support Team, CECC – Central Epidemic Command Center *For the moment, we are not witnessing the uncontained global spread of this virus, and we are not witnessing large-scale severe disease or deaths. Data source in worldwide referenced from Systems Science and Engineering at Johns Hopkins University [2]. Data source in Taiwan referenced from Taiwan Centers for Disease Control [3].

**Figure 1** summarizes the timeline of COVID-19 outbreak as of 28 April [1-3]. While 18 407 cases were confirmed in Iran with death toll of 1284 on 19 March, Italy surpassed China to become the country with the highest death toll (41 035 recorded cases, 3405 deaths). The British Prime Minister tested positive for COVID-19 on 27 March; and the UK recorded over 100 daily deaths for the first time (14 543 confirmed cases, 759 deaths on 28 March). Then Spain overtook China with the highest confirmed cases on 30 March; the United States overtook Italy to have the highest death toll in the world on 12 April (more than 2000 deaths in a single day); and Turkey overtook Iran to have the highest confirmed cases in Middle East on 18 April. France recorded the highest one-day increase (381 deaths) on 1 April with a total of infections exceeded 100 000 on 14 April. The confirmed cases (4003 within one day) increased to 103 228 in Germany on 8 April. Brazil became one of the worst-hit countries in Latin America with 17 857 cases and 941 deaths on 9 April. School closures, business shutdown, nationwide lockdown, borders closures, and shortage of medical supply and personal protective equipment have been reported throughout the world [1,2,4].

## CONTAINMENT IN TAIWAN

While COVID-19 is raging worldwide [4], Taiwan [5] – a non-WHO member, located 130 km from China with more than 400 000 of its 24 million citizens working there, and with almost three million Chinese visitors in 2019, which was predicted to be one of the countries that were most affected by the virus, has defied the expectation to have only 429 confirmed cases (343 imported, 55 indigenous, 31 Panshi fast combat support ship) and 6 deaths as of 28 April [3]. How Taiwan emerged as self-reliant from the experiences of 2003 severe acute respiratory syndrome (SARS) and 2009 H1N1 pandemic, has become a successful example during the COVID-19 pandemic. This article describes the measures that Taiwan took to prevent the spread of the novel coronavirus.

## AWARENESS OF SUSPECTED CASES

On 31 December 2019, epidemic prevention physicians of the Taiwan Centers for Disease Control (CDC) were alerted by seven cases with suspected atypical pneumonia from Wuhan, China of whom all had exposure history to the Huanan Seafood Market of Wuhan. Immediately on that day (31 December, 2019) Taiwan CDC sent an email to WHO International Health Regulations (IHR): “News resources today indicate that at least seven atypical pneumonia cases were reported in Wuhan, China. Their health authorities replied to the media that the cases were believed not SARS; however, the samples are still under examination, and the cases have been isolated for treatment” [3].

## GOVERNMENT ORGANIZATION AND RESPONSE

In response to potential pneumonia outbreak in Wuhan, Taiwanese government immediately activated enhanced border control and quarantine measures based on the possibility of human-to-human transmission assumption on 31 December, 2019. Two days after, Ministry of Health and Welfare Infectious Disease Control and Prevention Advisory Committee held the Influenza Control and Prevention Meeting in Preparedness to Potential Influenza Outbreak to formulate the first phase of prevention strategies [6]. In addition to the existing measure of onboard quarantine inspection, including fever screening of arriving passengers, suspected cases screening through history of travelling, occupation, contact and cluster (TOCC) inquiring, and health assessments conducting, all health care facilities should reinforce reporting severe cases of pneumonia among people who arrive in Taiwan from Wuhan. All health care workers should strictly adhere to standard precautions for preventing nosocomial infection, wearing N95 respirators as required while performing invasive medical procedures such as intubation and tracheostomy [3,6].

## CECC ACTIVATION AND EPIDEMIC CONTROL

To minimize the threat of the outbreak, on 20 January 2020, the Taiwan CDC activated the Central Epidemic Command Center (CECC) for Severe Special Infectious Pneumonia that composed of government agencies and medical experts/specialists to take charge of the domestic epidemic prevention and control, and to coordinate resources from across ministries and private stakeholders [3,6,7]. Since then, the CECC convened a daily press conference to update the latest epidemic, prevention measures and health guidelines. Such efforts included reporting criteria of all pneumonia cases, testing and quarantine procedures, preparation of pharmaceutical and medical supplies, capacity ensuring of isolation wards, and public health education (mask wearing, temperature checking, hand washing, avoiding eyes, nose, and mouth touching, environmental disinfection, etc.). Following identifying the first imported COVID-19 case returning from Wuhan on 21 January 2020, CECC raised travel notice level for Wuhan, China to Level 3 Warning. The confirmed case was treated in a negative pressure ward at the hospital, the close contacts were traced and the quarantined individuals were electronically monitored. In light of the ongoing outbreak, CECC has introduced guidelines for high-risk individuals regarding 1) self-health management (reported cases who have tested negative and met criteria for being released from isolation, or people under “COVID-19 Community-based Surveillance), 2) 14-day home quarantine (with travel history), and 3) 14-day home isolation (who had contact with confirmed cases), and provided quarantine sites with free meals and a US$30 compensation per day during the 14-day period. Furthermore, on 11 February an electronic health declaration system was established for returning travelers, allowing updated health care services. Starting from 19 March, home quarantine measures have been expanded to include arriving passengers from all countries. Taiwan designated 134 response and isolation hospitals, as well as 52 regional hospitals or medical centers for treatment of confirmed cases, to establish the Communicable Disease Control Medical Network (CDCMN) [3].

## TESTING, TRACING AND ISOLATING

According to the Communicable Disease Control Act, the Taiwan CDC classified COVID-19 as a Category 5 communicable disease on 15 January. People who meet the epidemiological criteria within 14 days: 1) History of traveling or living abroad, or contact with symptomatic (fever or other respiratory tract infection symptoms) individuals returning from abroad; 2) History of close contact with symptomatic suspected or confirmed case(s); 3) History of cluster related to confirmed cases are required to report to the CDC and placed for laboratory diagnosis. Furthermore, starting April, patients with pneumonia are required to be reported and subjected to testing. A total of 161 medical facilities were designated for collecting specimens. The laboratory testing capacity in Taiwan has reached 37 laboratories for 3900 cases per day. As of 9 May, 66 861 cases have been tested and 65 619 were excluded (as negative) with 440 laboratory-confirmed cases. People with three negative testing results were able to be released from isolation [3].

The community surveillance system has been implemented since 16 February for travelers to scan the QR Code with a Health Declaration and Home Quarantine E-System (Entry Quarantine System). People in 14-day home isolation were contacted twice a day by local health agencies to follow their health status. In collaboration with telecom companies, Taiwan launched an electronic security monitoring system to cellphone track people subjected to home quarantine/isolation [3,8]. The responsible civil affair bureau worker could receive a notification via SMS when the phone signal disappears, allowing the police to perform the location check. Violators could be fined or forcibly placed to the designated sites. Taiwan central and local governments provided consultation and support services for the assistance of transport arrangements, medical care arrangements and household services. Isolation hospitals, hotels and transportation arrangements for epidemic prevention were designated for home quarantined/isolated individuals. A Special Act for Prevention, Relief and Revitalization Measures for Severe Pneumonia with Novel Pathogens was adopted on 25 February to respond to the coming crisis [3].

## CHARTER PLANE OPERATION

To evacuate Taiwanese in Wuhan, CECC deployed a thorough onboard quarantine procedure. Individuals with symptoms were sent to negative pressure isolation wards directly upon arrival. All other evacuees were sent to designated quarantine sites by designated transport vehicles and placed under 14-day period of quarantine with their luggage disinfected. All related staff and health care workers participated in the charter plane operation wore personal protective equipment. For 19 Taiwanese passengers on board Diamond Princess, CECC deployed similar onboard quarantine and hospital isolation measures. Passengers with two negative testing results were subjected to central quarantine for 14 days and actively monitored [3].

## MASK REQUISITION AND TECHNOLOGY APPLICATION

Taiwan CDC began distributing surgical masks to the local convenience stores from 22 January 2020. To fulfill the needs from both health care workers and the public, Taiwanese government requisitioned all production of medical/surgical masks from domestic manufacturers from 31 January [3,7]. A name-based rationing system for mask purchase was launched on 6 February for the public with their National Health Insurance (NHI) cards. Masks were distributed to 6505 NHI contracted pharmacies, local social welfare bureaus and public health bureaus, with two masks per NHI card per week and each for the price of ~ US$0.17. Then an online ordering mechanism was added on 12 March, allowing the order of three masks per week through the NHI App. It increased to nine masks per NHI card every 14 days started from 9 April. The National mask team supplied up to 15.97 million masks in April. Meanwhile, the Taiwan CDC has provided Line @taiwancdc, translating AI technology through Medical Language Processing (MLP) for public interaction and consultation by cooperating with the health care division of technology innovator HTC, the DeepQ team.

## CULTURAL FACTORS

Different from the stigmatization in the West, the less negative perception of wearing a face mask in Taiwan (and Eastern Asia) has contributed to the “first line of defense” of COVID-19 [9]. For Taiwanese, it has become a cultural norm to wear a face mask due to various reasons, including: a courtesy to others when feeling sick, to keep face warm in winter, and as a protection from vehicle exhaust or air pollution. It is not implication for crime and security in the society. More importantly, from the SARS outbreak in 2003, Taiwanese government and citizens have learned how viral respiratory diseases can inflict and realized the impact of wearing a mask on reducing the risk of disease transmission, adopting mask wearing for COVID-19 was implemented quickly. People in Taiwan wear masks when taking mass transportation (metro, trains, buses and taxis), and going into the crowded public places. In addition, Taiwanese acknowledge scientific-based evidence, respect experts’ recommendations, and follow government’s policies at the Preparedness phase of the pandemic. The whole community voluntarily partnering with the government to create a network of databases with transparent information is another key to the success of “testing, tracing and isolating” strategy for fighting COVID-19 [8]. The Taiwanese government has been collaborating with developers and citizens to develop strategies and solutions at the online town hall (https://info.vtaiwan.tw/), where people can participate in the dialogue and the process of policy making and therefore, inspire the trust. The consensus of sharing digital data temporarily and of understanding how it is used have kept the virus from spreading in the community.

**Figure:**
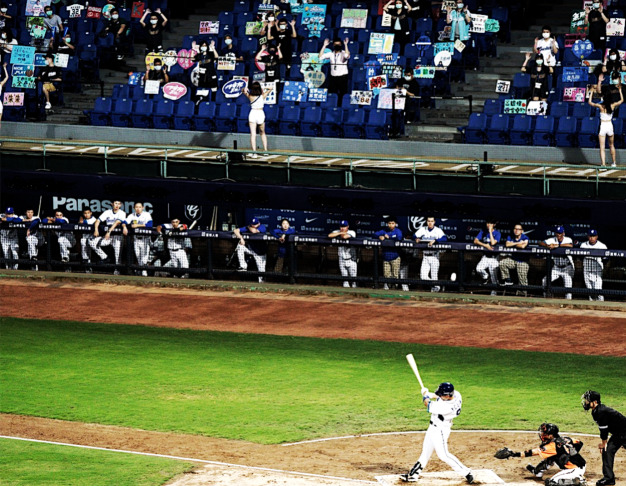
Photo: Taiwan’s Chinese Professional Baseball League played the first Opening Day ballgame of the 2020 season with audiences on 9 May. Available on Newtalk news, photo by Liang-I Chang, used with permission.

## CONCLUSION

Taiwan though excluded from membership in the WHO stands out in the COVID-19 pandemic as an example of successful prevention and outbreak control [5-7]. As of 9 May, while COVID-19 affected over 210 countries with a total of 3 937 813 confirmed cases and 274 898 deaths worldwide [2], Taiwanese society maintained routine daily life without long-term nationwide school closures or bushiness shutdown. Taiwan's baseball league was the first professional game to open the 2020 season on 11 April. Given its effective actions and managements, Taiwan had the capability to donate up to 17 million masks to the US, Europe, and diplomatic allies, and collaboratively work with the world [6,7]. It is the time to rethink how the world is tightly connected. Taiwan’s model of fighting COVID-19 set an example of how to effectively prevent or better response to the next public health crisis.

## References

[1] World Health Organization. Rolling updates on coronavirus disease. Coronavirus disease 2019 (COVID-19) situation report. Available: https://www.who.int/emergencies/diseases/novel-coronavirus-2019/events-as-they-happen. Accessed: 28 April 2020.

[2] Center for Systems Science and Engineering (CSSE). COVID-19 dashboard by the Center for Systems Science and Engineering (CSSE) at Johns Hopkins University (JHU). Available: https://gisanddata.maps.arcgis.com/apps/opsdashboard/index.html#/bda7594740fd40299423467b48e9ecf6. Accessed: 28 April 2020.

[3] Taiwan Centers for Disease Control. Press releases. Available: https://www.cdc.gov.tw/En/Bulletin/List/7tUXjTBf6paRvrhEl-mrPg?page=2. Accessed: 28 April 2020.

[4] Ravelo JL, Jerving S. COVID-19- a timeline of the coronavirus outbreak. Devex. Available: https://www.devex.com/news/covid-19-a-timeline-of-the-coronavirus-outbreak-96396. Accessed: 28 April 2020.

[5] Dewan A, Pettersson H, Croker N. As government fumbled their coronavirus response, these four got it right. Here’s how. CNN. Available: https://edition.cnn.com/2020/04/16/world/coronavirus-response-lessons-learned-intl/index.html. Accessed: 16 April 2020.

[6] Tsai I-W. President of Taiwan: How My Country Prevented a Major Outbreak of COVID-19. TIME. Available: https://time.com/collection/finding-hope-coronavirus-pandemic/5820596/taiwan-coronavirus-lessons/. Accessed: 16 April 2020.

[7] Chen S-C. Taiwan health minister: COVID-19 outbreak underscores importance of Taiwan’s inclusion in WHO. iPOLITICS. Available: https://ipolitics.ca/2020/04/17/covid-19-outbreak-underscores-importance-of-taiwans-inclusion-in-who-taiwan-health-minister/. Accessed: 17 April 2020.

[8] Kluth A. If We Must Build a Surveillance State, Let’s Do It Properly – As we develop new apps to track the coronavirus, the best model isn’t the U.S., China, Germany or South Korea. It’s Taiwan. Bloomberg Opinion. Available: https://www.bloomberg.com/opinion/articles/2020-04-22/taiwan-offers-the-best-model-for-coronavirus-data-tracking. Accessed: 22 April 2020.

[9] Griffiths J. Asia may have been right about coronavirus and face masks, and the rest of the world is coming around. CNN. Available: https://edition.cnn.com/2020/04/01/asia/coronavirus-mask-messaging-intl-hnk/index.html. Accessed: 20 April 2020.

